# Pathogenesis of Vitiligo: Integrating Immune and Non‐Immune Cell Crosstalk

**DOI:** 10.1111/1346-8138.70067

**Published:** 2025-12-01

**Authors:** Shintaro Inoue

**Affiliations:** ^1^ Cosmetic Health Science Laboratory Gifu Pharmaceutical University Gifu‐city Gifu Japan

**Keywords:** fibroblast, IFN‐γ, keratinocyte, resident memory T cells, vitiligo

## Abstract

Vitiligo is an acquired autoimmune disease characterized by depigmented macules resulting from melanocyte loss. It is a complex multifactorial disorder in which genetic predisposition is combined with environmental factors; however, its detailed etiology remains unclear. Although Janus kinase (JAK) inhibitors have recently emerged as a therapeutic option, the range of available molecularly targeted drugs is limited compared to those for atopic dermatitis or psoriasis, necessitating an urgent elucidation of its pathogenesis. The pathogenesis of vitiligo is centrally mediated by cytotoxic CD8^+^ T cells (CTLs) specific for melanocyte antigens and their production of interferon‐gamma (IFN‐γ). In recent years, however, the involvement of other immune cells, such as resident memory T cells and regulatory T cells, innate immune cells, and non‐immune cells including keratinocytes and fibroblasts has also garnered attention. Furthermore, pathogenic alterations are also present in clinically normal‐appearing non‐lesional skin, indicating that this tissue is “primed” for disease development. This finding supports a paradigm shift toward viewing vitiligo as a systemic disease rather than a localized skin disorder. Herein, this review summarizes the current knowledge on the factors leading to the onset and progression of non‐segmental vitiligo, while also briefly addressing segmental vitiligo.

## 1 Introduction

Vitiligo is an acquired, intractable depigmenting disorder characterized by the loss or reduction of melanocytes (MCs), resulting in the absence of melanin pigment. It is a complex, multifactorial autoimmune disease that develops when environmental factors act on a genetic predisposition. Although the detailed etiology remains unknown, it has long been thought that MC‐targeting CTLs and the interferon‐gamma (IFN‐γ) they produce play a central role in the pathogenesis of vitiligo [1, 2, 3, 4]. However, in recent years, evidence has accumulated implicating other immune system components, such as tissue‐resident memory T cells (TRMs) [5], immunosuppressive regulatory T cells (Tregs) [6], and innate immune cells like natural killer (NK) cells and innate lymphoid cells (ILCs) [7]. Furthermore, non‐immune cells, including keratinocytes (KCs) [8], and fibroblasts (FBs) [9], are now recognized as critical contributors to the pathogenesis, maintenance, and recurrence of the disease.

With improvements in living conditions, the age of onset for vitiligo has been increasing, leading to a greater recognition of the importance of various environmental factors as triggers for its development [10]. Environmental factors such as reactive oxygen species (ROS) and infections can first damage MCs and KCs, and the subsequent activation of the innate immune system and dendritic cells (DCs) is involved in the onset, maintenance, and progression of vitiligo. Understanding the roles of these cell types, as well as the damage‐associated molecular patterns (DAMPs), pathogen‐associated molecular pattern molecules (PAMPs), cytokines, and chemokines they release, is crucial for the diagnosis and treatment of vitiligo [11, 12].

Recently, it has been noted that, compared to the skin of healthy individuals, the seemingly healthy non‐lesional skin of patients with vitiligo already exhibits subclinical immune activation, cellular fragility, and an imbalance between immune activation and suppression [13, 14]. This implies that the non‐lesional skin of patients with vitiligo exists in a precarious “unstable equilibrium” between destructive and regulatory forces, rendering it susceptible to disease initiation by various triggers. In other words, vitiligo is not a localized disease confined to the site of occurrence but a systemic condition, and treatment strategies must take this into account. The complex interplay between these immune and non‐immune cells, even in clinically unaffected skin, forms the basis for the emerging view of vitiligo as a systemic disorder.

This review summarizes recent findings regarding the mechanisms of onset and the pathogenic factors leading to the maintenance and progression of non‐segmental vitiligo (described as vitiligo), while also briefly discussing segmental vitiligo.

## 2 Factors in the Onset and Progression of Vitiligo

**FIGURE 1:**
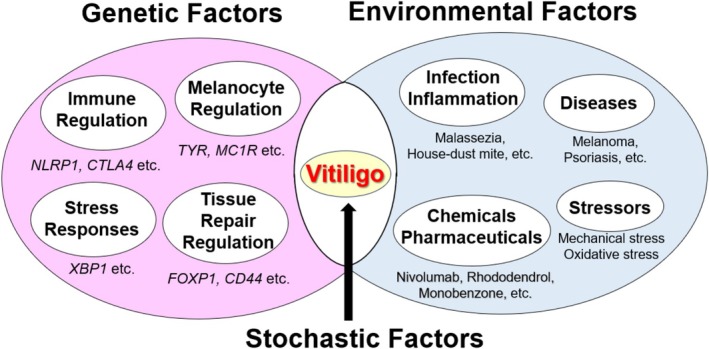
The multifactorial etiology of vitiligo. The onset and progression of vitiligo are depicted as a convergence of three distinct yet interacting categories of factors. Genetic factors confer a predisposition through variants in genes related to immune regulation and MC function. Environmental factors, such as infections, chemical exposures, and mechanical stress, act as triggers that initiate cellular damage. Stochastic factors, including epigenetic modifications and metabolic imbalances, are believed to be the final determinants that disrupt homeostasis and precipitate disease in genetically susceptible individuals exposed to environmental triggers.

Vitiligo onset and progression are driven by a combination of genetic, environmental, and stochastic factors (Figure 1). The involvement of genetic factors is evident from several facts: (1) first‐degree relatives of patients have a 5–6 times higher risk of developing the disease; (2) the risk for the second twin in monozygotic twins is 23 times higher if the first has vitiligo; (3) vitiligo often co‐occurs with other autoimmune diseases; (4) genes related to innate and adaptive immunity have been identified as risk genes; and (5) vitiligo does not follow Mendelian inheritance patterns and involves multiple susceptibility loci (multifactorial) [1, 2, 3].

Simultaneously, the involvement of the innate and adaptive immune systems in vitiligo onset is supported by findings such as: (1) early‐onset cases are associated with a haplotype in a transcriptional regulatory region that amplifies HLA class II expression; (2) innate immune cells and external/internal stimuli (chemicals, PAMPs, DAMPs, etc.) are involved in onset; and (3) genome‐wide association studies (GWAS) have revealed that about half of the vitiligo susceptibility genes encode immunoregulatory proteins [3, 11, 12].

However, the fact that environmental factors are also involved is inferred from observations such as: (1) only 23% of monozygotic twins develop vitiligo concordantly; (2) the sum of all identified genetic risks accounts for only 25% of the total genetic risk; and (3) the disease shows a bimodal age of onset, with only pediatric cases (one‐third of the total) being associated with the HLA class II expression control locus, while the remaining patients develop it as adults [2, 3]. An analysis of the age of onset in vitiligo patients over a 62‐year period from 1951 showed a shift from pediatric to adult onset, with the average age increasing linearly during a 30‐year period that coincided with environmental improvement measures such as air and water purification and sun protection, suggesting that environmental factors contributed to the aging of vitiligo onset [10].

Environmental factors that induce or exacerbate vitiligo include diseases such as melanoma and psoriasis [15]; infectious and inflammatory stimuli like house‐ dust mites and Malassezia [11, 16]; immune checkpoint inhibitors (ICIs) such as nivolumab (NIV) and chemicals like rhododendrol [17, 18]; and mechanical or oxidative stress [19, 20]. However, even when both genetic and environmental factors are present, vitiligo does not necessarily develop. It is believed that stochastic factors, such as epigenetic changes and complex metabolic imbalances, combine to trigger the onset of vitiligo (Figure 1) [21].

Detailed investigation of the histological identity and mechanisms of vitiligo formation in cases where the cause is clear, such as NIV‐induced vitiligo and rhododendrol‐induced vitiligo (RIV) [17, 18], is beneficial for elucidating the mechanisms of vitiligo of unknown cause.

## 3 Is Non‐Lesional Vitiligo Skin Equivalent to Healthy Skin?

**FIGURE 2:**
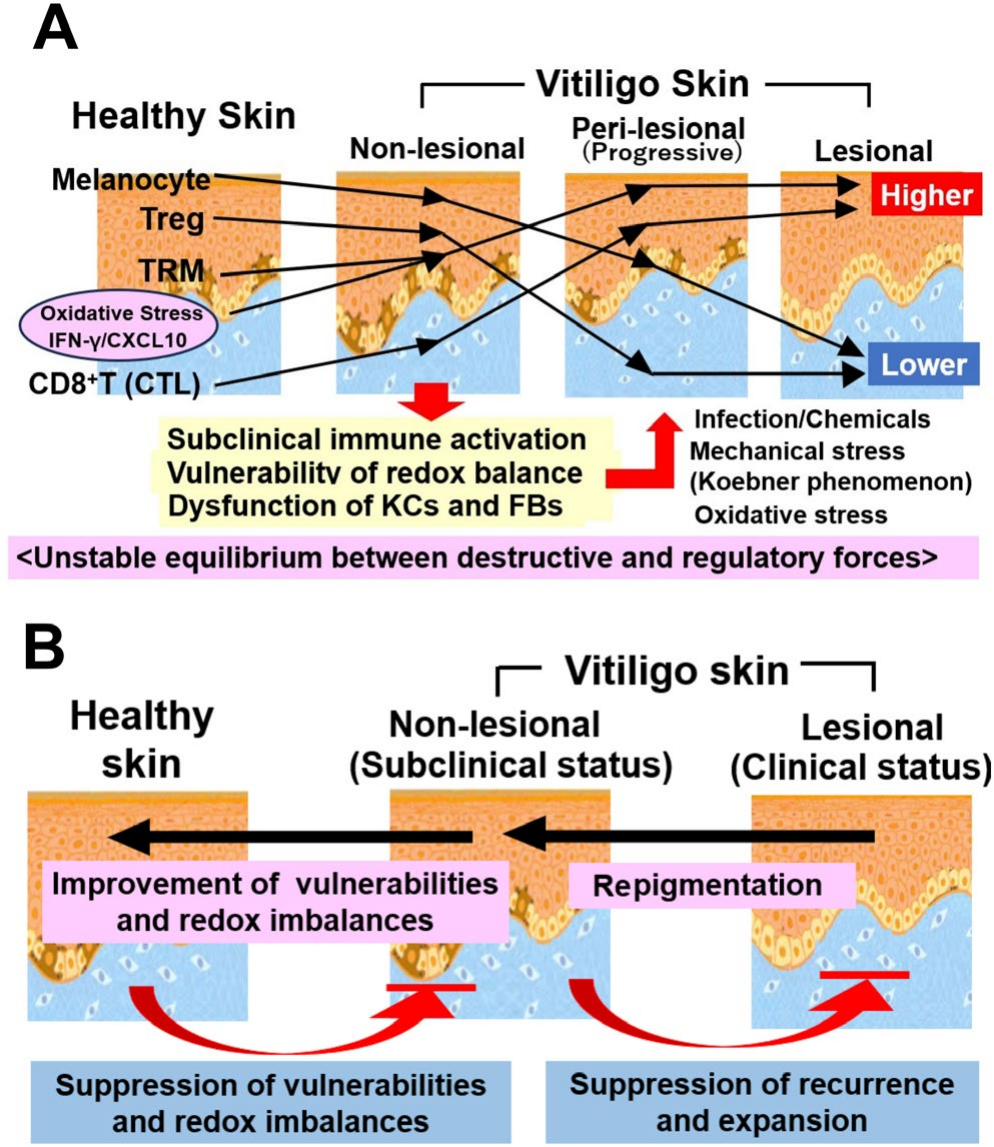
The “primed’ state of non‐lesional skin and its therapeutic implications. (A) The unstable equilibrium in non‐lesional skin. Compared to healthy skin, the clinically normal‐appearing skin of patients with vitiligo exists in a subclinically inflamed or “primed” state. This is characterized by a subtle decrease in MCs and an increase in cytotoxic CD8^+^ T cells and TRMs, coupled with compromised Treg function. This creates a precarious equilibrium between destructive and regulatory forces, which can be easily tipped toward depigmentation by various triggers (e.g., mechanical stress), leading to the Koebner phenomenon. (B) A paradigm shift in therapeutic strategy. The recognition of non‐lesional skin's vulnerability compels a shift from a localized treatment model, focused solely on repigmenting visible lesions, to a systemic approach. The new paradigm aims to restore immune homeostasis across the entire skin, thereby not only treating active lesions but also suppressing the subclinical inflammation in non‐lesional areas to prevent disease progression and recurrence.

Research into the pathology and etiology of vitiligo has traditionally focused on how lesional and borderline areas differ from non‐lesional areas. Particularly during the onset, progression, and repigmentation phases of vitiligo, changes at the border between lesional and non‐lesional areas were thought to be directly linked to treatments aimed at preventing spread and inducing repigmentation. However, recently, a debate has emerged as to whether the seemingly healthy non‐lesional skin is truly healthy compared to the skin of healthy individuals [13, 14]. An immunohistochemical analysis of 25 cases each of healthy skin and non‐lesional vitiligo skin showed no statistically significant difference in MC and CD8^+^ T cell counts, but a comparison of median values revealed a decrease in MCs and an increase in CD8^+^ T cells in non‐lesional areas (Figure 2A) [22]. An increase in CD8^+^ T cells in the dermis of non‐lesional skin, similar to that in lesional skin, has been confirmed in patients with active vitiligo [23]. In the epidermis of non‐lesional vitiligo skin, IFN‐γ positive signals tended to increase, and the T‐cell homing signal CXCL10 was significantly increased compared to healthy skin. Interestingly, cultured KCs derived from non‐lesional skin showed a markedly increased production of CXCL10 and IL‐1β upon scratching (mechanical stress) compared to KCs from healthy skin [24].

One of the biggest challenges in vitiligo treatment is the high frequency of recurrence after treatment discontinuation. In many cases, lesions reappear in the same location where pigment had once recovered. This immunological memory is now attributed to TRMs, a specialized T cell subset that persists long‐term within peripheral tissues such as the skin, rather than recirculating through lymphoid organs [24]. TRMs are present in both non‐lesional and healed areas of vitiligo skin. Upon re‐recognizing autoantigens, they can quickly re‐ignite an immune response by exerting their direct MC‐attacking capabilities, constantly exposing non‐lesional areas to the risk of vitiligo recurrence and progression [25]. In healthy skin, CD4^+^ Tregs suppress the excessive activation of CD8^+^ T cells and TRMs, maintaining immune homeostasis. However, in vitiligo patients, this Treg‐mediated control mechanism is thought to be compromised [26, 27]. The homeostasis of non‐lesional areas is precariously maintained by a dynamic and unstable equilibrium between these opposing forces. This equilibrium is extremely fragile, and environmental triggers, such as trauma leading to the Koebner phenomenon [28] or psychological stress [29], can disrupt this balance, tipping it toward effector‐dominant responses and readily precipitating clinical depigmentation (Figure 2A).

In other words, vitiligo is not a local disease confined to the site of occurrence but a systemic one, where subclinical immune activation, cellular fragility, and an imbalance between immune memory and regulation are already present in non‐lesional areas. This paradigm has profound implications for the development of novel therapeutic strategies, shifting the focus from treating visible lesions to restoring systemic immune homeostasis (Figure 2B).

## 4 Nivolumab‐Induced Vitiligo

Among ICIs, nivolumab (NIV), an anti‐PD‐1 antibody, prevents the loss of CD8^+^ T cell function by blocking the binding of the immune checkpoint ligand PD‐L1/2 expressed by cancer cells to PD‐1 expressed on CTLs. According to reports of drug‐induced vitiligo side effects from the US Food and Drug Administration's Adverse Event Reporting System since 2016, the top three drugs with the most reported adverse events of vitiligo were all ICIs [30]. Compared to a total of 84 cases of vitiligo caused by TNF‐α inhibitors, NIV was associated with a strikingly high number of 330 cases.

**FIGURE 3:**
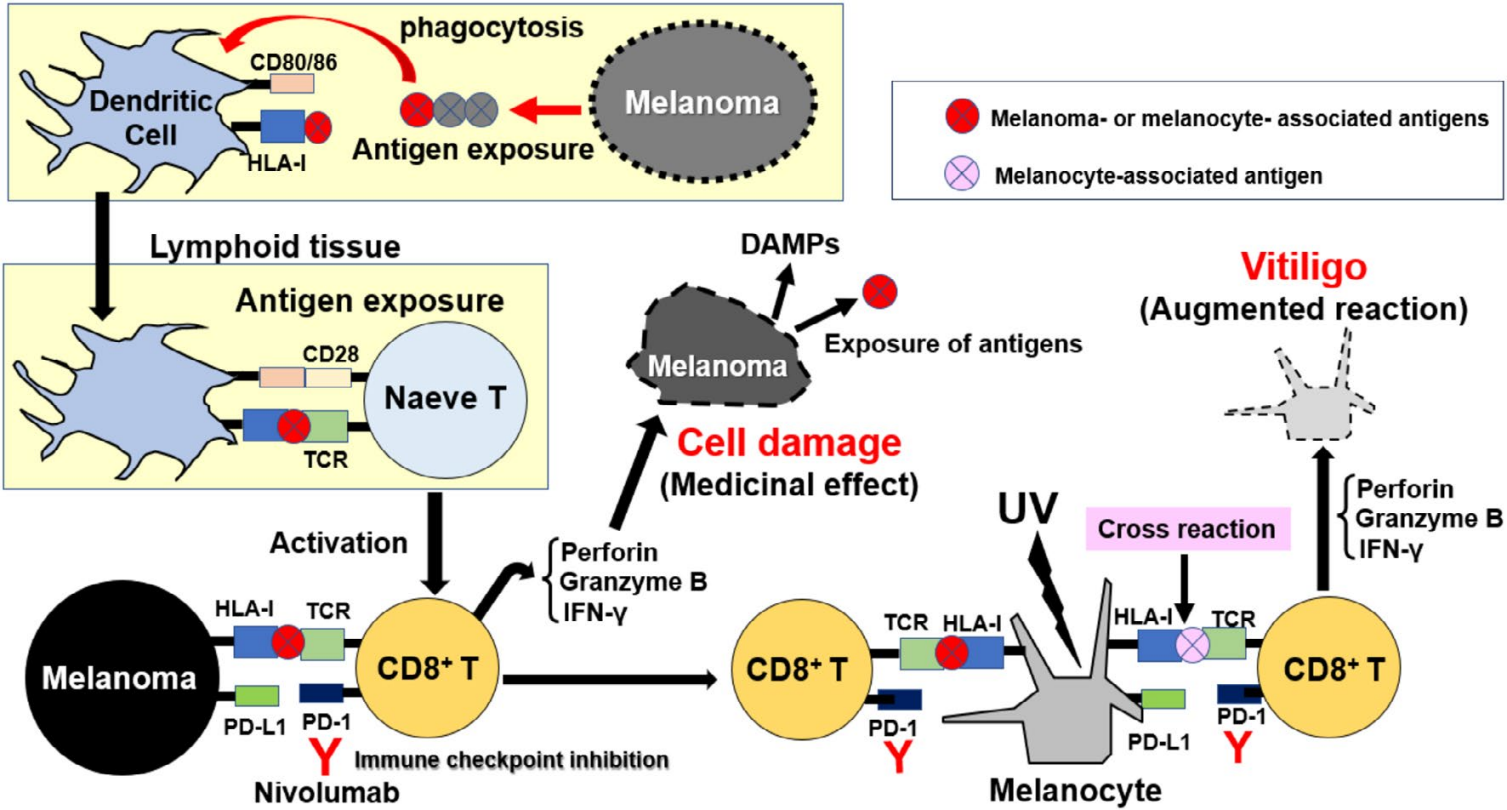
Pathomechanism of nivolumab‐induced vitiligo. This figure illustrates a model for drug‐induced autoimmunity. Nivolumab, an immune checkpoint inhibitor, blocks the interaction between PD‐1 on CD8^+^T cells and PD‐L1 on other cells, including melanoma cells. This blockade unleashes the cytotoxic activity of T cells against melanoma. However, these activated T cells, primed against melanoma‐associated antigens, can cross‐react with similar self‐antigens expressed by normal MCs. This cross‐reactivity is often precipitated in UV‐exposed areas, where MC stress may enhance antigen presentation. The subsequent attack by perforin‐ and granzyme B‐releasing CD8^+^ T cells leads to MC destruction and the clinical manifestation of vitiligo. This process is initiated by dendritic cells that phagocytose melanoma antigens and present them to naive T cells in lymphoid tissues.

Nivolumab‐induced vitiligo is characterized by: (1) a specific pattern of multiple, punctate depigmented lesions; (2) no association with a personal or family history of autoimmune diseases; and (3) a correlation between serum CXCL10 levels and skin infiltration by IFN‐γ/TNF‐α‐producing CXCR3^+^ CD8^+^ T cells [17]. Interestingly, in all examined cases, the vitiligo lesions were confined to sun‐exposed areas and were not accompanied by the Koebner phenomenon. This suggests that activated CTLs mediate MC destruction and subsequent lesion formation in response to melanoma antigens or cross‐reactive autoantigens presented exclusively on UV light‐exposed melanocytes (Figure 3). This is consistent with reports that the response rate in patients with advanced melanoma, the therapeutic response rate to NIV is significantly higher in those who develop vitiligo [31]. It can be inferred that the presence of melanoma (antigen presentation) is essential for the onset of vitiligo during ICI application, suggesting a commonality with the onset mechanism of vitiligo seen in patients with nevi or melanoma.

## 5 Rhododendrol‐Induced Vitiligo (RIV)

Rhododendrol (RD) is a cosmetic ingredient that suppresses melanin production, and RD‐induced leukoderma (RIL) was observed in approximately 2.4% of estimated users of cosmetics containing it [18, 32, 33]. Of these, 96% had leukoderma consistent with the application site, and about 80% showed incomplete depigmentation. Many cases recovered after discontinuing the use of the cosmetics, but about 0.1% of users experienced an expansion of leukoderma to non‐application sites, which was refractory and clinically indistinguishable from vitiligo (RIV). This refractory leukoderma was considered to be the result of RIL transitioning to RIV due to unknown background factors [18, 33].

Previous studies have revealed that the mechanism of MC‐specific cell death and reversible RIL formation involves the generation of oxidative metabolites such as RD‐quinone, as RD acts as a substrate for tyrosinase [32], and an increase in oxidative stress due to the depletion of intracellular glutathione in MCs [34]. On the other hand, RD oxidative metabolites activate intracellular NRF2 to confer resistance to oxidative stress [34], and the activation of autophagy allows cells to escape cell death [35]. Therefore, it was thought that RIL develops when the complex balance of the oxidative stress response is disrupted [18]. This corresponds to the environmental and stochastic factors in Figure 1, and it is believed that when genetic factors are added, a vitiligo‐like RIV pathology emerges. Indeed, GWAS analysis identified the T‐cadherin gene (*CDH13*) as an RIV susceptibility gene, and it was shown that reduced expression of CDH13 increases the RD sensitivity of MCs, and is involved in the enhancement of tyrosinase and the suppression of BCL‐2 and BCL‐XL expression [36]. In the future, to further clarify the relationship with the etiology of vitiligo, it is necessary to investigate in detail the possibility that non‐immune cells such as KCs and FBs, as well as immunological factors, are involved in the onset of RIV.

## 6 Cellular Contributors to Vitiligo Pathogenesis, Maintenance, and Progression

### 6.1 Melanocyte (MC)

High levels of superoxide dismutase and low levels of catalase have been observed in vitiligo skin, and the resulting hydrogen peroxide can easily pass through the MC membrane, causing cytotoxicity [37, 38]. As a result, MCs from patients become more sensitive to oxidative stress. The mitochondria of MCs in non‐lesional areas are already altered, and ROS have accumulated [39]. Detailed histological observation in patients with stable vitiligo confirmed the presence of melanin pigment in both lesional and non‐lesional areas, a small number of MCs in the lesional area, and the existence of “floating MCs” detached from the basal layer [40]. Degenerated MCs in the lesional area contained melanosomes, melanin granules, and autophagosomes. Single‐cell RNA sequencing analysis of cells from lesional areas also confirmed the presence of MCs and their characteristic gene expression [26].

**FIGURE 4:**
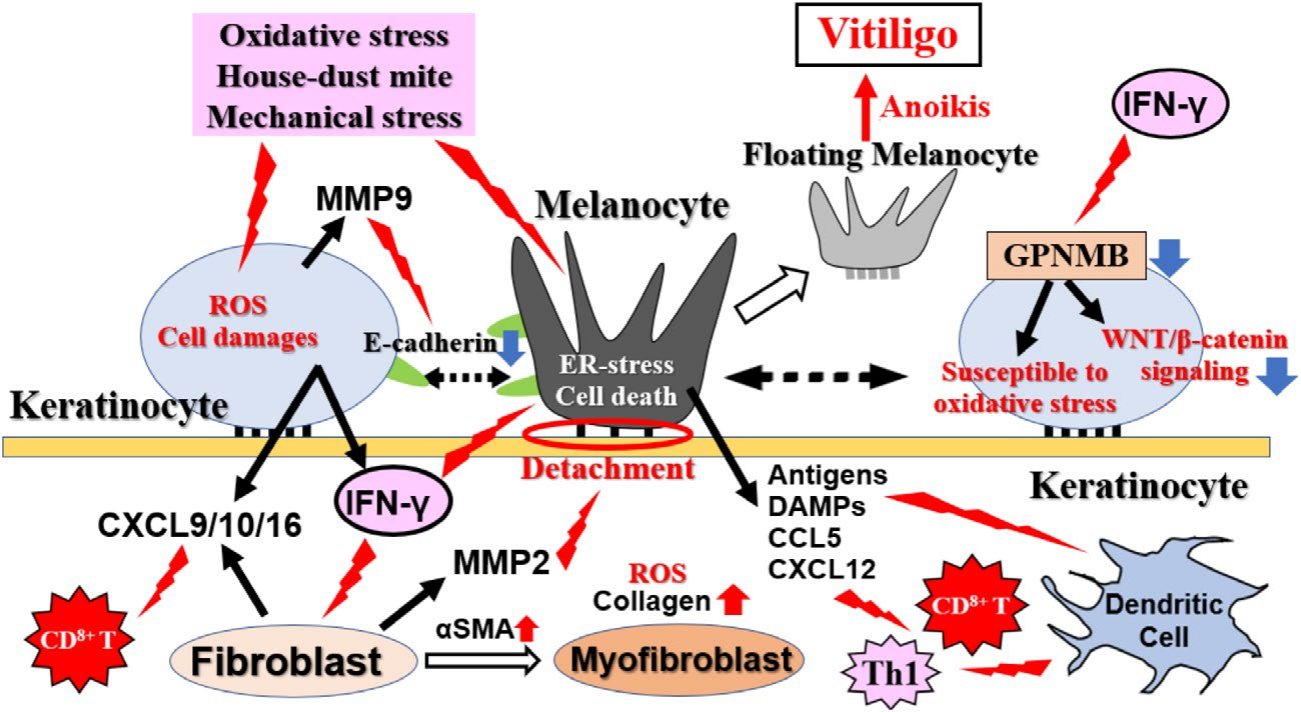
Crosstalk between KCs, FBs, and MCs in vitiligo pathogenesis. This schematic details the non‐immune cell interactions that drive MC detachment and amplify immune responses. Initial stressors damage MCs, causing them to release antigens and DAMPs. These signals stimulate surrounding KCs and FBs. Stimulated KCs, under the influence of IFN‐γ, downregulate the adhesion molecule E‐cadherin and GPNMB, while upregulating MMP‐9. This concerted action disrupts MC adhesion, leading to the formation of “floating MCs” and anoikis. Concurrently, activated FBs differentiate into myoFBs (α‐SMA positive), produce MMP‐2 which degrades the basement membrane, and secrete chemokines (e.g., CXCL9/10) that recruit immune cells, thereby creating a self‐perpetuating inflammatory loop.

**FIGURE 5:**
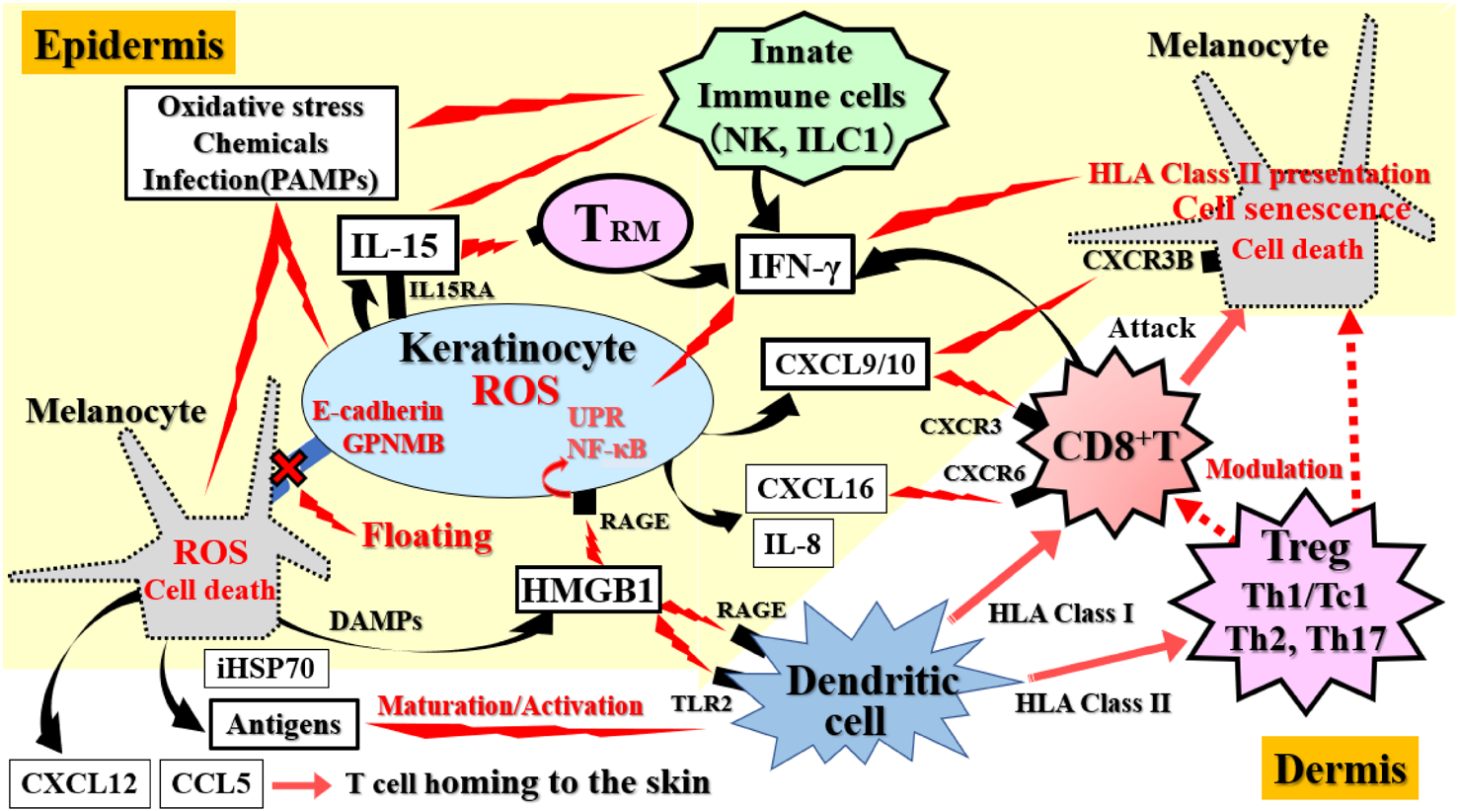
The immune cascade driving MC destruction. This figure outlines the key immunological events in vitiligo pathogenesis. (1) Initiation: Environmental stressors induce MC and KC damage, leading to the release of MC‐specific antigens, DAMPs, chemokines, and cytokines. (2) Innate Immune Activation: Stressors and KC‐derived IL‐15 stimulate innate immune cells (NK cells, ILC1s), triggering IFN‐γ production. (3) Antigen Presentation: DCs mature in response to these signals, process MC antigens, and migrate to lymph nodes to prime autoreactive T cells. (4) Adaptive Immune Effector Phase: Antigen‐specific CD8^+^ T cells are recruited to the epidermis via a chemokine gradient. Upon antigen recognition, CD8^+^ T and TRM cells release IFN‐γ and cytotoxic molecules, leading to MC apoptosis. The maintenance and activation of TRMs are critically dependent on IL‐15, which is trans‐presented by KCs. (5) Impaired Regulation: This autoimmune attack is exacerbated by a local reduction or functional impairment of Tregs.

Furthermore, it has been reported that the formation of floating MCs detached from the basement membrane involves a decrease in E‐cadherin levels in MCs and KCs, E‐cadherin degradation by KC‐derived matrix metalloproteinase (MMP)‐9, and destruction of the basement membrane by FB‐derived MMP‐2 (Figure 4) [41]. MCs damaged by oxidative or ER stress present or release MC‐associated antigens, DAMPs (such as HMGB1, iHSP70, and calreticulin), and chemokines (such as CXCL12 and CCL5), which are involved in stimulating KCs and DCs and in the homing of T cells to the skin (Figures 4 and 5) [11, 41, 42].

### 6.2 Keratinocyte (KC)

In the lesional skin of stable vitiligo, hyperkeratosis, basal vacuolization, acanthosis, KC ballooning, and spongiosis are observed, along with a decrease in E‐cadherin expression and an increase in apoptotic cells. Various degenerative changes in KCs and MCs, disorganization of desmosomes between KCs, and a lack of intracellular melanosomes in KCs are also seen [40], suggesting that not only MCs but also KCs are abnormal in vitiligo lesions. These abnormalities are thought to be a mixture of the processes and consequences of vitiligo formation, such as the effects of UV light due to melanin deficiency, the influence of PAMPs and DAMPs, or the effects of cytokines like IFN‐γ.

**FIGURE 6:**
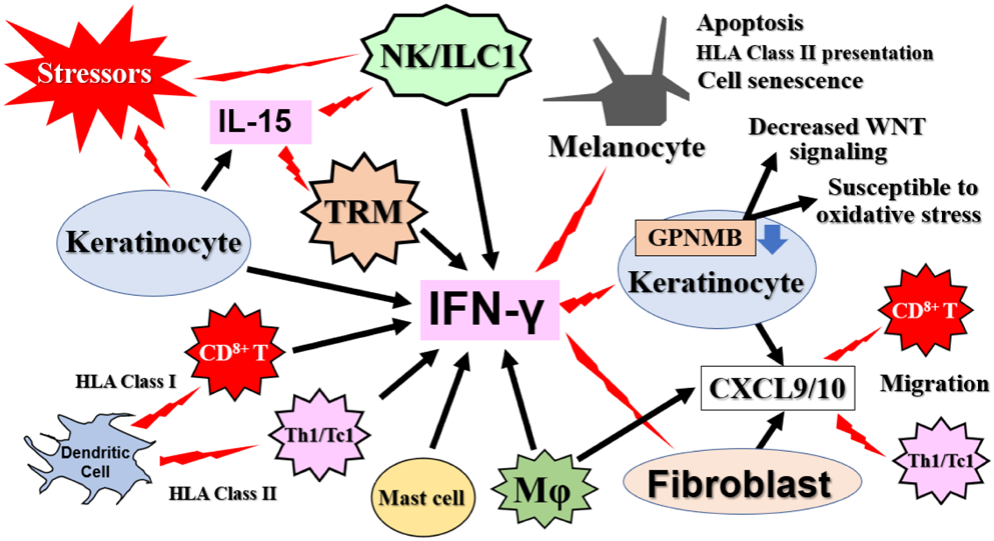
The central role of the IFN‐γ axis in the vitiligo pathogenic network. IFN‐γ is depicted as the master cytokine orchestrating a positive feedback loop that sustains vitiligo pathogenesis. Multiple cell types, including KCs, innate immune cells (NK, ILC1), Mφs, mast cells, and adaptive immune cells (CD8^+^ T and TRM cells), serve as sources of IFN‐γ in response to initial triggers. IFN‐γ then acts on primarily KCs and FBs, stimulating them to produce the CXCR3‐ligand chemokines CXCL9 and CXCL10. These chemokines recruit additional IFN‐γ‐producing CD8^+^T cells into the skin. Furthermore, IFN‐γ downregulates KC GPNMB expression, leading to the increased apoptosis and fragility of KCs. This establishes a self‐amplifying cycle of MC destruction.

During the progression of vitiligo, CXCL9/10 secreted from KCs upon IFN‐γ stimulation recruits CTLs to the epidermis, inducing the progression of vitiligo (Figures 4 and 5) [8, 26, 43]. HMGB1 stimulates KCs via pattern recognition receptors such as the receptor for advanced glycation end‐products (RAGE) and Toll‐like receptor 2 (TLR2), inducing the expression of inflammatory factors like CXCL16, which stimulates DC maturation and skin CD8+ cell infiltration (Figure 5) [11, 44]. KCs also secrete IL‐15 upon stimulation, and in lesional KCs, the IL‐15 receptor subunit (IL15RA) is highly expressed, allowing them to bind IL‐15 and present it to TRMs and ILCs [45]. Thus, KCs play a crucial role in the maintenance and activation of TRMs and ILCs (Figures 4, 5, 6).

Interestingly, the protease antigen Der p1 from house‐dust mites has been detected in patient skin, and when cultured KCs were stimulated with this antigen, they were reported to produce not only CXCL10 and CXCL16 but also significantly more IFN‐γ compared to KCs from healthy individuals (Figures 4 and 5) [16]. Furthermore, in situ cultured skin, antigen dose‐dependent induction of MMP‐9 production and MC detachment from the basement membrane were observed. These findings demonstrate that environmental factors, such as house‐dust mite antigens, can induce KCs to become a noncanonical source of IFN‐γ, while simultaneously promoting MC detachment through MMP‐9 induction (Figures 4, 5, 6). Additionally, in the epidermis of patients with vitiligo, the conversion of kynurenine to kynurenic acid is enhanced in KCs, resulting in the activation of aryl hydrocarbon receptor (AHR) and the induction of CXCL10 production [46].

Not only in stable and progressive vitiligo but also in non‐lesional areas, subclinical pathological changes in KCs have been reported [24]. The epidermis of non‐lesional areas already shows thickening and parakeratosis compared to healthy skin, and CXCL10 production is elevated. Interestingly, cultured KCs from non‐lesional areas induced the production of CXCL10 and IL‐1β upon scratch stimulation (Figure 2A).

In the basal layer of lesional areas in vitiligo and RIV patients, the expression of the membrane protein GPNMB, which is involved in cell adhesion, survival, and stress response, was found to be lost, and its expression tended to be lower in the borderline area compared to healthy skin [47]. Furthermore, GPNMB expression in cultured KCs was suppressed in a JAK/STAT‐dependent manner by IFN‐γ stimulation (Figures 4 and 6). Decreased expression of GPNMB in KCs increased sensitivity to oxidative stress, with suppression of the PI3K/AKT and WNT/β‐catenin pathways [48]. Additionally, the GPNMB ectodomain, released from the membrane by ADAM10, contributed to the oxidative stress resistance of MCs [49]. Collectively, these results implicate the loss of GPNMB in multiple pathogenic processes, including the increased apoptosis and fragility of KCs, the heightened oxidative stress sensitivity of MCs, and the formation of “floating” MCs. Intriguingly, this pathway may also contribute to the observed reduction in skin cancer risk among individuals with vitiligo (Figure 4).

In summary, many findings from patient epidermis and cultured human KCs indicate that KCs are deeply involved in vitiligo pathogenesis and maintenance through non‐immune, innate immune, and adaptive immune pathways.

### 6.3 Fibroblast (FB)

FBs from non‐lesional vitiligo areas produce ROS and have myofibroblast‐like properties (Figure 4). In cultured FBs from lesional areas, the production of IL‐6 and HGF is enhanced, which reduces E‐cadherin production in MCs [50]. It has also been reported that IFN‐γ‐responsive FBs are present in the borderline area where MCs exist during progressive vitiligo, and they produce high levels of CXCL9/10 to recruit CD8^+^ T cells [9]. Furthermore, the density of IFN‐γ‐responsive FBs in a given area correlates positively with the incidence of vitiligo. In the non‐lesional skin of patients with active vitiligo, an increase in CD8^+^ T cells in the dermis, similar to that in lesional skin, was observed, which was suggested to be the result of enhanced CXCL9/10 production by FBs stimulated by IFN‐γ produced from KCs or other cells in response to external stimuli (Figures 4 and 6) [23].

It has also been suggested that FBs in progressive vitiligo lesions produce MMP‐2, which degrades the basement membrane and contributes to the formation of floating MCs (vitiligo formation) [51]. On the other hand, FBs in stable lesions were activated and showed a myofibroblast‐like phenotype, with enhanced collagen production and antioxidant capacity (Figure 4) [52]. These reports indicate that FBs may play a major role in vitiligo formation and maintenance. Further research is needed to consider them as therapeutic targets.

### 6.4 Innate Immune Cells (Innate Lymphoid Cell; ILC1, Natural Killer (NK) Cell)

Innate immunity is based on the ability of pattern recognition receptors to detect PAMPs from pathogens and DAMPs from damaged cells. NK cells and ILC1s, which contribute to the innate response, produce high levels of IFN‐γ under stress conditions such as ROS and DAMPs (Figures 5 and 6) [7]. Both cell types are increased in the blood and non‐lesional skin of patients with vitiligo, and abnormally activated NK cells expressing granzyme B (GZMB) have been found in lesional areas, suggesting they are important in the initial steps leading to MC loss in lesions [54]. Monobenzone, which induces chemical leukoderma, induces an MC‐specific immune response characterized by NK cell activation and macrophage (Mφ) infiltration [55]. These findings suggest that ILC1s and NK cells are the initial source of IFN‐γ, a characteristic cytokine of vitiligo, and are involved in MC damage either directly or via T cells.

### 6.5 DC

After MC damage due to stress, MC antigens are processed and presented by DCs. Activated DCs locally release cytokines to induce T‐cell activation and recruitment to the skin, and in local lymph nodes, they induce the recruitment of CD8^+^ T cells, bridging innate and adaptive immunity (Figure 3). HMGB1 and HSP70 secreted from MCs in vitiligo skin, and calreticulin presented on the membrane, promote DC maturation [11, 42]. iHSP70 increases the number of DCs that release inflammatory signals to initiate an immune response (Figures 4, 5, 6) [12, 56]. CXCL16 from HMGB1‐stimulated KCs also stimulates DC maturation and skin CD8^+^ infiltration [11]. Monocyte‐derived DCs (moDCs) that infiltrate vitiligo skin during inflammation activate CD8^+^ effector memory T cells (Tems) that secrete large amounts of IFN‐γ upon IFN‐α stimulation [57]. These findings indicate that DCs contribute to the induction of autoimmunity through both MC‐derived antigen processing and innate immune activation, and also play a crucial role in bridging to adaptive immunity [43].

### 6.6 Cytotoxic CD8+ T Cell

The marginal tissue of vitiligo lesions shows infiltration mainly of CD8^+^ T cells, which are preferentially located at the dermal‐epidermal junction adjacent to MCs [58]. Even in non‐lesional areas, subclinical levels of CD8^+^ T cells have been confirmed compared to healthy skin (Figure 2A) [22, 23]. CXCL9/10 released from IFN‐γ‐responsive FBs in the marginal area contributes to the infiltration of CD8^+^ T cells (Figures 4, 5, 6). Stressed MCs activate MC‐specific CD8^+^ T cells, leading to MC destruction [59], and the number of CD8^+^ T cells at the lesion border correlates with disease progression and severity [60]. Autoreactive CD8^+^ T cells infiltrating the margin eliminate MCs in vitro, whereas isolated CD4^+^ T cells lack this activity [61]. In the skin of the vitiligo margin and lesion, CD8^+^ T cells secrete high levels of IFN‐γ and GZMB [26]. These findings provide strong evidence that in vitiligo, CD8^+^ T cells produce IFN‐γ and damage MCs (Figures 3, 4, 5, 6). In response, JAK inhibitors (e.g., ruxolitinib) that interfere with the IFN‐γ signaling pathway by inhibiting Janus kinase (JAK) 1 and JAK2 have been developed as topical treatments for vitiligo [62].

### 6.7 CD8 ^+^ Resident Memory T Cell (TRM)

CD8^+^ TRMs, which play a central role in immune memory, include central memory T cells, Tems, and TRMs, which differentiate in peripheral tissues and remain there for a long time [61]. In the skin, they patrol the epidermis and papillary dermis and are rapidly activated upon encountering nonself‐antigens [63], In vitiligo, recurrence at the same site after repigmentation is observed [64], and since TRMs are also IFN‐γ‐producing cells, they are important for the recurrence and maintenance of vitiligo (Figures 5 and 6).

TRMs express CD69, CD103 (Eβ7 integrin), CD49a, and CD44 (hyaluronan binding protein) on their cell surface [65, 66]. The margins of stable and active vitiligo lesions show high infiltration of TRMs expressing both CD69 and CD103 compared to normal human skin [67, 68]. CD69 prevents TRMs from exiting peripheral tissues [69], CD103 binds to E‐cadherin in the epidermis [68], and CD44 binds to hyaluronan in the KC intercellular spaces [70], suggesting that TRMs have a very high affinity for the epidermal layer.

It has also been reported in mouse models that TRMs remaining in the skin play an important role in inducing Tems and maintaining vitiligo [71]. The level of cytotoxic activity due to GZMB and perforin production by CD8^+^ TRMs in vitiligo skin was not different from healthy controls [67], suggesting that the damaging activity of TRMs depends mainly on inflammatory cytokines such as IFN‐γ, and that they mediate long‐term disease maintenance and recurrence through the cytokine‐mediated recruitment of circulating T cells. IL‐15, which is upregulated in vitiligo lesions, is essential for the maintenance of these pathogenic TRMs and enhances their effector functions [45, 66]. Consequently, the IL‐15/IL‐15R axis, particularly the trans‐presentation of IL‐15 by keratinocytes, represents a highly promising therapeutic target for eliminating autoimmune memory and achieving durable disease remission (Figures 5 and 6).

TRMs have been detected in non‐lesional areas as well as at the margins [25, 72], indicating that inactivated TRMs in non‐lesional areas can form new lesions. The homeostasis of non‐lesional areas is precariously maintained by a dynamic and unstable equilibrium between opposing forces, where Tregs suppress the full activation of TRMs, but cannot completely quell the subclinical inflammation maintained by TRMs (Figure 2).

### 6.8 CD4 ^+^ Regulatory T Cell (Treg)

Tregs are one type of immunosuppressive cell, comprising about 5%–10% of peripheral blood CD4^+^ T cells in healthy individuals, and exhibit a CD4^+^, CD25^+^, and FOXP3^+^ phenotype [73]. They play a role in suppressing immune responses against self, but an increase or activation of Tregs in aging or patients with cancer weakens anti‐tumor immune responses, while a decrease or reduced activity of Tregs increases the risk of autoimmune diseases [6, 74].

In patients with vitiligo, Treg activity is normal, but their numbers are reduced in lesional areas. Single‐cell RNA sequencing analysis of cells from lesional and marginal areas also showed a decrease in the number of Tregs per CD8^+^ T cell [6, 26, 75]. These results suggest that a reduction in their localization to the skin, rather than a decline in Treg function, contributes to the pathology of vitiligo. On the other hand, there are reports showing no significant decrease in Treg numbers in vitiligo sites [76, 77], reduced ability of peripheral Tregs to suppress the proliferation and activation of CD8^+^ T cells in vitro [78], and Th1‐like Tregs generation, which show high IFN‐γ and low IL‐10 production under the influence of the inflammatory microenvironment in patients with vitiligo [79]. Detailed research on how a decrease, functional decline, or functional change in Tregs contributes to the onset of vitiligo is important for considering therapies that improve Treg function.

### 6.9 CD4 ^+^ Helper T Cells (Th1, Th2, Th17)

CD4^+^ helper T cells are T cells that are activated when exogenous antigen peptides are presented on HLA class II molecules of antigen‐presenting cells, and include Th1, Th2, Th17, and Tregs (Figure 5). A comparative analysis of serum from vitiligo patients and healthy individuals showed that innate pro‐inflammatory cytokines (IL‐1β, IL‐15, TNF‐α, etc.) were predominant in patients with vitiligo, and the ratio of Th1 cytokines (IFN‐γ, TNF‐β, etc.) to Th2 cytokines was higher [80]. It was also suggested that Th2 cytokines play a protective role. Regarding Th17, the production of IL‐17 by Th17 cells in the peri‐lesional region was shown to be comparable to the amount secreted from healthy skin, and indeed, an evaluation study of the anti‐IL‐17A agent secukinumab for the treatment of vitiligo confirmed that IL‐17 or Th17 does not play a direct role in vitiligo pathogenesis (Figure 5) [67, 81, 82].

The expression of HLA class II on MCs upon IFN‐γ stimulation [83] and the promotion of Th1 cell homing by CXCL12 and CCL5 production from MCs [84] suggest an interaction between MCs and CD4^+^ T cells in vitiligo (Figures 4 and 5).

### 6.10 Other Cells

M1 Mφs have been reported as a source of IFN‐γ and CXCL9/10 (Figure 6) [26, 85]. In single‐cell RNA analysis of cells from vitiligo skin, Mφs were present in both lesional and non‐lesional areas and expressed CXCL9/10 at higher levels than in non‐lesional areas [26]. An increase in Mφs in vitiligo lesions and peripheral blood, and a decrease in M2 Mφs in lesions have been reported [86, 87].

The above report also notes an increase in resting mast cells in lesional areas. Human mast cells have NK‐like markers and functions [88], and connective tissue‐type mast cells amplify the Th1‐type immune response via STAT4 [89]. They may play a role as IFN‐γ‐producing cells (Figure 6) [90], and conversely, they may have a role as suppliers of histamine [91] and SCF [92], which promote melanin production. Further research on mast cells in vitiligo is warranted.

## 7 The Overlapping Pathogenesis of Segmental Vitiligo (SV) With Nonsegmental Vitiligo (Non‐SV)

SV has a unilateral distribution, with a combination of additional features such as the blaschkoid pattern, irregular margins, and islands of pigmented macules within the lesions [93]. SV is traditionally thought to arise from non‐immune mechanisms. The leading hypotheses include the neural theory, which posits that neurochemical mediators released from nerve endings cause MC destruction, and the somatic mosaicism theory, which suggests that a post‐zygotic mutation renders a localized population of MCs vulnerable to damage [94]. However, this distinction is becoming less rigid, as recent studies have identified a similar inflammatory infiltrate, rich in MC‐specific CD8 + T cells, in early SV lesions, akin to that seen in non‐SV. These findings have led to the proposal of a ‘convergence theory,’ [95] suggesting that a localized trigger in SV (e.g., a fragile mosaic patch) may incite a secondary autoimmune response that shares effector pathways with non‐SV, thus explaining cases of mixed vitiligo and unifying the disease spectrum under a common framework of immune‐mediated MC loss.

## 8 Vitiligo as A Disorder of Impaired Repair Mechanisms Against Melanocyte Damage and Cell Death

In the cases of RIV and ICI‐induced vitiligo, the trigger for vitiligo onset is first the damage to MCs by chemicals or UV light exposure, but damage and repair are repeated daily in the skin. The fact that this repetition, combined with genetic and stochastic factors such as stress response ability, damage repair ability, and immune response ability, leads to vitiligo‐like RIV with an incidence of about 0.1% is the cause. The repair of MCs also includes the supply of epidermal MCs from melanocyte stem cells (MSCs) or mesenchymal stem cells (MSCs) [96, 97]. Similarly, vitiligo can be understood as a disorder of impaired repair against persistent damage and cell death of MCs. Research to attract or supply MSCs or MSCs to regenerate MCs lost during the onset or recurrence of vitiligo is anticipated.

## 9 Conclusion

In vitiligo, it has become clear that environmental factors such as oxidative stress and infection act as triggers, leading to crosstalk between the innate immune system, the adaptive immune system, and non‐immune cells such as KCs and FBs, mediated by mediators like IFN‐γ, IL‐15, and CXCL9/10, resulting in a pathological state where MCs are constantly damaged. These mechanistic insights have directly informed the development of novel therapeutic strategies, such as JAK inhibitors [98], which target the downstream signaling of key cytokines including IFN‐γ and IL‐15.

On the other hand, advances in etiological and pathological research have revealed that in the non‐lesional areas of vitiligo patients, subclinical immune activation, cellular fragility, and an imbalance between immune memory and regulation are already present (Figure 2A). These findings compel a conceptual shift in therapeutic strategy: from treating vitiligo as a localized disorder confined to lesional areas to managing it as a systemic disease that involves the entire skin organ and requires therapies aimed at restoring immune homeostasis (Figure 2B). In addition to JAK inhibitors, the development of new therapeutic agents such as phosphodiesterase‐4 inhibitors and AHR agonists is progressing [99, 100], and detailed trials to correct the fragile imbalance in non‐lesional areas (including choice between topical and oral administration, timing and duration of treatment, and combination with phototherapy) are anticipated in the future. Future research exploring systemic influences, such as the gut‐skin axis and cellular metabolic pathways, may further illuminate the initial triggers that disrupt this fragile cutaneous equilibrium and unveil novel targets for intervention.

## Conflicts of Interest

The author declares no conflicts of interest.

## Data Availability

Data sharing not applicable to this article as no datasets were generated or analyzed during the current study.
